# Hepadnavirus DNA Is Detected in Canine Blood Samples in Hong Kong but Not in Liver Biopsies of Chronic Hepatitis or Hepatocellular Carcinoma

**DOI:** 10.3390/v14071543

**Published:** 2022-07-15

**Authors:** Yan Ru Choi, Min-Chun Chen, Maura Carrai, Francesca Rizzo, Yingfei Chai, May Tse, Ken Jackson, Vito Martella, Joerg Steiner, Patricia A. Pesavento, Julia A. Beatty, Vanessa R. Barrs

**Affiliations:** 1 Centre for Animal Health and Welfare, Jockey Club College of Veterinary Medicine and Life Sciences, City University of Hong Kong, Kowloon Tong, Hong Kong SAR 518057, China; yrchoi@cityu.edu.hk (Y.R.C.); mcarrai@cityu.edu.hk (M.C.); vanessa.barrs@cityu.edu.hk (V.R.B.); 2 Gastrointestinal Laboratory, Department of Small Animal Clinical Sciences, Texas A&M University, College Station, TX 77843, USA; mcchen@cvm.tamu.edu (M.-C.C.); jsteiner@cvm.tamu.edu (J.S.); 3 Jockey Club College of Veterinary Medicine and Life Sciences, City University of Hong Kong, Kowloon Tong, Hong Kong SAR 518057, China; francesca.rizzo@cityu.edu.hk; 4 Department of Veterinary Clinical Sciences, Jockey Club College of Veterinary Medicine and Life Sciences, City University of Hong Kong, Kowloon Tong, Hong Kong SAR 518057, China; yingchai@cityu.edu.hk; 5 CityU Veterinary Diagnostic Laboratory, City University of Hong Kong, Kowloon Tong, Hong Kong SAR 518057, China; maypy.tse@cityu.edu.hk; 6 School of Veterinary Medicine, UC Davis, Department of Pathology, Microbiology, and Immunology, Davis, CA 95616, USA; kajackson@ucdavis.edu (K.J.); papesavento@ucdavis.edu (P.A.P.); 7 Department of Veterinary Medicine, University of Bari, 70010 Valenzano, Italy; vito.martella@uniba.it

**Keywords:** carcinoma, hepatocellular, cats, dogs, *Hepadnaviridae*, hepatitis B virus, hepatitis, chronic

## Abstract

Chronic hepatitis and hepatocellular carcinoma (HCC) caused by the hepadnavirus hepatitis B virus (HBV) are significant causes of human mortality. A hepatitis-B-like virus infecting cats, domestic cat hepadnavirus (DCH), was reported in 2018. DCH DNA is hepatotropic and detectable in feline blood or serum (3.2 to 12.3%). Detection of HBV DNA has been reported in sera from 10% of free-roaming dogs in Brazil, whereas 6.3% of sera from dogs in Italy tested positive for DCH DNA by real-time quantitative PCR (qPCR). If DCH, HBV, or another hepadnavirus is hepatotropic in dogs, a role for such a virus in the etiology of canine idiopathic chronic hepatitis (CH) or HCC warrants investigation. This study investigated whether DCH DNA could be detected via qPCR in blood from dogs in Hong Kong and also whether liver biopsies from dogs with confirmed idiopathic CH or HCC contained hepadnaviral DNA using two panhepadnavirus conventional PCRs (cPCR) and a DCH-specific cPCR. DCH DNA was amplified from 2 of 501 (0.4%) canine whole-blood DNA samples. A second sample taken 6 or 7 months later from each dog tested negative in DCH qPCR. DNA extracted from 101 liver biopsies from dogs in Hong Kong or the USA, diagnosed by board-certified pathologists as idiopathic CH (*n* = 47) or HCC (*n* = 54), tested negative for DCH DNA and also tested negative using panhepadnavirus cPCRs. This study confirms that DCH DNA can be detected in canine blood by qPCR, although at a much lower prevalence than that reported previously. We identified no evidence to support a pathogenic role for a hepadnavirus in canine idiopathic CH or HCC.

## 1. Introduction

*Hepadnaviridae* is a family of small, hepatotropic DNA viruses that infect mammals (genus *Orthohepadnavirus*), birds (*Avihepadnavirus)*, frogs, and reptiles (*Herpetohepadnavirus*), and fish (genus Metahepadnavirus and Parahepandaviruses) [1]. Hepatitis-B virus (HBV), the most extensively characterized hepadnavirus, is a major cause of liver disease in humans [2]. Chronic HBV infection causes an immune-mediated hepatitis that can progress to cause death from cirrhosis or hepatocellular carcinoma (HCC) [3].

Hepatitis-B-like viruses are increasingly being identified in diverse hosts [4,5,6,7,8], but our understanding of their pathogenic potential has not kept pace, being largely limited to hosts of interest as animal models of HBV. Of these, infection of the Eastern woodchuck (*Marmota monax*) with Woodchuck hepatitis virus (WHV) most predictably causes chronic hepatitis (CH) and HCC resembling HBV-associated disease [9].

The discovery of domestic cat hepadnavirus (DCH) [6] provides a new imperative to understanding the pathogenicity of hepadnaviruses, that of safeguarding companion animal health, with strategies developed to combat HBV presenting potential reverse translational benefits for pets. DCH has been detected in cats in Australia, Italy, Thailand, Malaysia, the UK, and the USA. Molecular prevalence in blood or serum ranges from 3.2 to 12.3% [6,10,11,12,13,14], and persistent infection of individual cats over several months is reported [15]. Phylogenetic analysis of complete DCH genomes shows close clustering on a branch distinct from other mammalian hepadnaviruses [6,10,12,13]. Evidence for a link between DCH infection and liver disease in cats is increasing; DCH DNA was detected via PCR in liver biopsies from 43% (6/14) of cats with chronic hepatitis and 28% (8/29) of cats with hepatocellular carcinoma but not in cats with other liver diseases or normal feline liver (0/39) [11]. Histologic features and viral distribution in DCH-associated cases resembled those seen with HBV-associated diseases. In addition, increased activity of serum ALT, a marker of hepatocellular damage, has been identified as a risk factor for DCH detection [12,13].

Whether a pathogenic hepadnavirus infects domestic dogs is not known. HBV DNA was detected in 10% (19/189) of sera from wild domestic dogs in Brazil [16], and in Italy, sera from 40/635 dogs (6.3%) undergoing routine laboratory testing tested positive using a qPCR designed to detect DCH [17]. A complete genome of the dog-derived hepadnavirus shared 98% nucleotide homology with DCH, and a similarly high homology (97.7 to 98.7%) was found between canine-derived hepadnaviral sequences [17]. Evidence of an antibody response was also detected; 13/20 DCH qPCR positive canine sera tested positive for antibodies recognizing DCH core antigen on Western blot [17]. The possibility that dogs are susceptible to infection with a hepadnavirus requires further investigation, not least because of the potential for hepadnavirus-associated diseases in this major companion species.

Two significant canine health problems are candidates for hepadnavirus involvement; canine idiopathic chronic hepatitis (CH) and HCC. Idiopathic CH is a common diagnosis in veterinary practice accounting for 12% of first opinion cases in the UK [18]. Idiopathic CH shares histological features with human viral hepatitis, but evidence for an infectious etiology remains elusive [19,20,21]. A study including 38 biopsies from dogs with a diagnosis of chronic hepatitis failed to identify hepadnavirus DNA using degenerate panhepadnavirus (consensus) primers [19]. Consensus primers that target highly conserved regions are useful tools to discover novel viruses that are closely related to known viruses [22]. Since Boomkens et al. (2005) published their results, the World Small Animal Veterinary Association standards for the classification of liver disease have been published [23]. Hence, a new study is warranted, one that could also take advantage of sequence data made available in the interim to update panhepadnavirus PCR design. HCC is the most common primary liver tumour in dogs, as it is in humans where HBV causes over half of all cases [24]. To the authors’ knowledge, screening of canine HCC for hepadnavirus involvement has not yet been reported.

This study had two aims: firstly, to determine whether hepadnavirus DNA can be detected in blood samples from dogs in Hong Kong using qPCR designed to detect DCH and, secondly, to investigate biopsies from confirmed cases of idiopathic CH and HCC diagnosed in Hong Kong and the USA for the presence of hepadnavirus DNA.

## 2. Materials and Methods

### 2.1. Samples

Two independent sample sets were obtained and characterized.

#### 2.1.1. Canine Blood Samples

To investigate whether DCH DNA could be detected in dogs in Hong Kong, residual diagnostic whole-blood samples collected with owner consent during a routine investigation of dogs treated at City University Veterinary Medical Centre (CUVMC), Hong Kong, were obtained and stored at −80 °C until processing for qPCR (approved by the Animal Ethics Committee of City University, Hong Kong, approval number A-0696). The CUVMC caseload includes first opinion and referral cases.

The final sample set comprised 501 blood samples, based on a minimum sample size of 457, using an estimated prevalence of 5% (+/−2%) [17] with a confidence level set at 95% [25]. Samples were collected between 1 March 2021 and 31 May 2021, inclusive. Data on age, sex, neuter status and breed were collected when available and analyzed using descriptive statistics.

#### 2.1.2. Canine Liver Biopsies

To investigate biopsies from confirmed cases of idiopathic CH and HCC for the presence of DNA of DCH or a novel hepadnavirus, archived formalin-fixed, paraffin-embedded (FFPE) liver samples diagnosed by board-certified veterinary pathologists were obtained from three institutions; CityU Veterinary Diagnostic Laboratory, Hong Kong, the University of California at Davis, USA and Texas A&M University, College Station, TX, USA. The number of each sample type submitted from each institution was recorded.

### 2.2. qPCR of Canine Whole-Blood-Derived DNA

DNAs were extracted from 100 µL of samples using the DNeasy Blood and Tissue Kits (QIAGEN GmbH, Hilden, Germany) with an elution volume of 50 µL, as described previously [26]. The real-time quantitative PCR (qPCR) designed to detect DCH, which was also used to investigate the prevalence of hepadnavirus DNA in canine sera in a previous study was used, as described [10]. The primers and probe, targeting a 132 bp sequence in the polymerase gene, are presented in Table 1. A plasmid was prepared by cloning a 1.4 kb fragment of the polymerase region of Australian DCH reference strain AUS/2016/Sydney with a TOPO XL-2 PCR cloning kit (Thermo Fisher Scientific, Waltham, MA, USA), according to the manufacturer’s instructions. Tenfold dilutions of the plasmid, representing 10^1^ to 10^9^ copies of DNA/10 μL of template, were used to generate a standard curve for absolute viral DNA quantification. All standards and template DNAs were run in triplicate with results presented as mean values. Molecular grade water was used as a negative control. Quantitative PCR was carried out in 25 μL reactions containing 12.5 μL of master mix (IQ Supermix; Bio-Rad Pacific Limited, Hong Kong), 600 nM of primers FHBV-for and FHBV-rev, 200 nM of probe, and 10 µL of template in Tris–EDTA (TE), using 5–100 ng template DNA/reaction. Thermal cycling consisted of activation of iTaq DNA polymerase at 95 °C for 3 min and 42 cycles of denaturation at 95 °C for 10 s and annealing–extension at 60 °C for 30 s. Assays were run using a CFX96 touch system (Bio-Rad Pacific, Ltd., Quarry Bay, Hong Kong), with a cutoff of R-squared set at 0.980 an efficiency at 90–110%. Data were analyzed with CFX Maestro software. A sample was defined as positive if at least 3 copies of DCH DNA per reaction were detected in at least two of three replicates. Virus load in positive samples was expressed as copies per mL of blood by obtaining the mean copies per reaction of positive wells and multiplying by 50.

### 2.3. Conventional PCR of Canine-Liver-Derived DNA

DNA was extracted from 10 µm sections of formalin-fixed paraffin-embedded (FFPE) liver tissue using DNeasy Blood and Tissue Kits (QIAGEN GmbH, Hilden, Germany), as described previously [26]. To confirm the integrity of the extracted DNA template, cPCR was performed for the ubiquitous gene encoding glyceraldehyde 3-phosphate dehydrogenase (GAPDH) [6,27]. All the extracted DNA samples generated the expected 80 bp product on gel electrophoresis.

Liver-derived DNAs were investigated using a DCH-specific cPCR [6] and two nested, degenerate panhepadnavirus PCRs [28,29]. PCR primers and cycling conditions are presented in Table 1. Each reaction contained 1 uL of template DNA, DreamTaq™ Hot Start Green DNA Polymerase (Thermo Fisher Scientific, Cleveland, OH, USA), dNTP (Thermo Fisher Scientific, Graciuno, Vilnius, Lithuania) at a final concentration of 200 μM, and a final primer concentration of 300 nM for the virus-specific PCR and 500 nM for panhepadnavirus PCRs. For panhepadnavirus cPCRs, 1 μL of the PCR product from the first round was used as template for the second round. No-template (molecular-grade water) and positive controls (DCH-positive whole-blood-derived DNA) were included in all cPCR assays. Products were resolved using 1.5% agarose gel electrophoresis.

**Table 1:** Oligonucleotides used in this study.

Primer Set/Rationale for Use	Target	Name	Purpose	Sequence
DCH qPCR [10]/ to investigate canine blood-derived DNA for DCH	Polymerase gene (132 bp)	FHBV-for	For	CGTCATCATGGGTTTAGGAA
		FHBV-rev	Rev	TCCATATAAGCAAACACCATACAAT
		FHBV-prob	Probe	[FAM]TCCTCCTAACCATTGAAGCCAGACTACT [QSY]
DCH-specific cPCR [6]/ to investigate DNA from canine liver lesions for DCH	Core protein gene (258 bp)	Hgap-F	For	CTAGAATGGCTACATGGGTTAG
		Hgap-R	Rev	GTGCTCTGATAACCGTATGCTC
Panhepadnavirus Adapted from [6]/ to investigate DNA from canine liver lesions for DCH for any known or novel hepadnavirus	Highly conserved region of the polymerase gene (1st round: 493 bp 2nd round: 258 bp)	HBV-pol-F1	1st for	TAGACTSGTGGTGGACTTCTC
		HBV-pol-R1	1st rev	CATATAASTRAAAGCCAYACAG
		HBV-pol-F2_2	2nd for	CCTCATCTTCTTGTTGGTTC
		HBV-pol-R2	2nd rev	AGTRAAYTGAGCCAGGAGAAAC
Panhepadnavirus [29]/ to investigate DNA from canine liver lesions for any known or novel hepadnavirus	Highly conserved region of the polymerase gene (1st round: 504 bp 2nd round: 306 bp)	HBV_266os	1st for	GTGGTGGAYTTCTCWCARTT
		HBV_763oa	1st rev	CCCCAAWACCANRTCATCCATA
		HBV_386is	2nd for	GATGTRTCTGCGGCGTTYTATC
		HBV-pol-R2	2nd rev	AGTRAAYTGAGCCAGGAGAAAC
GAPDH cPCR [26]/ to confirm integrity of extracted DNA	Coding sequence of canine GAPDH GenBank: AB038240.1 (80 bp)	GAPDH-For	For	AAGGCTGAGAACGGGAAAC
		GAPDH-Rev	Rev	CATTTGATGTTGGCGGGATC

## 3. Results

### 3.1. Detection of Hepadnavirus DNA in Canine Blood

In total, 2 of 501 canine whole-blood-derived DNA samples (0.4%) tested positive for hepadnavirus DNA using qPCR. The characteristics of the study population are presented in Table 2. One qPCR-positive sample, from an 11-year-old female neutered Pomeranian, contained 5.0 × 10^4^ copies per mL of blood (mean Ct 30.53). DNA from a repeat extraction of the same sample also tested qPCR-positive, with a viral load of 1.8 × 10^4^ copies per mL of blood. An additional blood sample from the same dog taken 7 months later tested negative. The second qPCR-positive dog, an 8-and- a-half year-old female neutered Pomeranian, contained 2.0 × 10^3^ copies per mL of blood (mean Ct 35.1). On follow-up testing of DNA reextracted from the same sample, viral load was 2.0 × 10^3^ copies per mL of blood. A second blood sample taken 6 months later from the same dog tested negative. DCH-specific cPCR of qPCR-positive samples from both dogs was attempted to obtain sequence data, but both samples tested negative.

### 3.2. Hepadnavirus DNA Not Detected in Canine Liver Lesions

**Figure 1:**
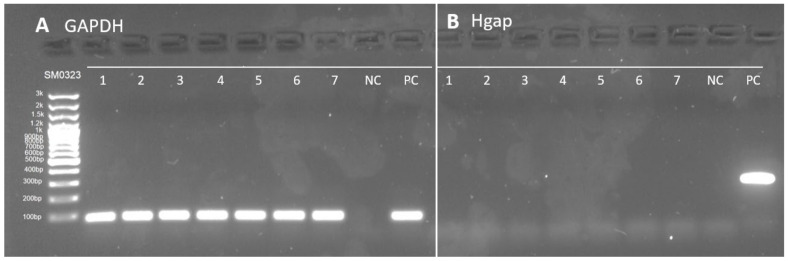
Representative results of conventional (c)PCR testing of DNA extracted from liver biopsies from dogs with canine idiopathic chronic hepatitis or hepatocellular carcinoma. Samples 1 to 7 all tested (**A**) positive for GAPDH and (**B**) negative for domestic cat hepadnavirus. All 101 samples tested negative on 3 cPCRs for hepadnavirus DNA. NC = negative control, PC = positive control.

DNA from 101 liver biopsies, comprising 47 cases of idiopathic CH (13 from HK, 18 from UC Davis, and 16 from TAMU), 54 cases of HCC (21 from HK, 20 from UC Davis, and 13 from TAMU), and 6 normal liver samples (UC Davis) were tested with DCH-specific cPCR and 2 additional consensus primer panhepadnavirus PCR tests. All samples tested negative for hepadnavirus sequences (Figure 1).

## 4. Discussion

DCH DNA was detected in whole blood from two dogs from Hong Kong, supporting the findings of a recent study conducted in Italy [17]. The PCR prevalence of hepadnavirus detection in dogs in Hong Kong was 0.4%, in contrast to that reported in Italy where the prevalence was 6.3% using the same qPCR assay in a similar population, i.e., owned dogs undergoing laboratory testing. It is not possible to know whether the divergence in observed prevalence represents a regional effect. The provenance of the affected animals is unknown since deidentified samples were used in this study. However, the importation of dogs into Hong Kong has been common, accompanying an internationally mobile population in line with the region’s status as an economic and education hub. Additionally, in contrast to the Italian study [17], which used serum DNA as template, we used whole-blood DNA as template so it is possible that this methodological difference contributed to the apparent difference in prevalence. Nonetheless, marked geographic variation in prevalence is well-established for other hepadnaviruses. For example, WHV is endemic in Eastern woodchucks in mid-Atlantic states and North Carolina, where antigen prevalence varies from 12.5 to 16.9% [30,31], but markers of WHV infection are almost absent in other areas, including central New York State [32,33].

The relationship between hepadnavirus sequences detected in dogs and DCH, which is known to infect cats on at least several continents, is not yet clear. We were unable to obtain sequence data from the two canine samples that tested hepadnavirus-positive with qPCR, precluding phylogenetic analysis. A full-length genome and a partial sequence obtained from dogs in Italy using rolling circular DNA amplification were phylogenetically indistinguishable from DCH [17]. This finding is intriguing since hepadnaviruses typically infect only one or a small number of closely related hosts, so DCH would not necessarily be expected to also infect domestic dogs, which are classified in a separate genus from cats, within the Order Carnivora.

The negative qPCR finding in a second sample that had been collected several months later from both dogs that originally tested positive in this study may indicate transient infection. In the study by Diakoudi et al. (2022), 10 of the 13 dogs testing seropositive for DCH-core antibodies had an IgM response, either alone (2/13), or in combination with IgG (8/13). It is possible that DCH infection in dogs is rapidly cleared by the immune response before infection can be established in the liver. Alternatively, copy number may fluctuate since, in HBV-infected patients, virus load can fluctuate by several hundred-fold even in stable patients [34]. Future studies might include canine necropsy liver specimens in addition to whole blood or serum to provide hepadnavirus-positive tissue for sequencing since orthohepadnaviruses are hepatotropic, and the liver serves as a reservoir of viral DNA in chronic infections.

The absence of detectable hepadnavirus sequences in biopsies from cases of idiopathic CH and HCC using either a DCH-specific cPCR or panhepadnavirus cPCRs makes it unlikely that a hepadnavirus has a role in the development of these liver diseases in dogs. In comparison to pathology induced by human and rodent hepadnaviruses, canine idiopathic CH and hepatocellular carcinoma are strong candidates for hepadnavirus involvement [35]. Pathogenic mechanisms in hepadnaviral diseases include immune-mediated liver damage, as well as indirect and direct oncogenesis [34]. HBV-associated inflammatory and neoplastic lesions contain viral DNA that is readily amplified via cPCR [36,37].

Alternatively, CH and HCC may be heterogeneous diseases, and it is conceivable that hepadnaviruses are associated with a subset of lesions that were not sampled here. In this regard, it may be worthwhile to test liver tissue from dogs with CH or HCC in other geographic regions or in different populations than the ones studied here.

Finally, a role for a hepadnavirus in acute canine hepatitis has not been ruled out. Duck hepatitis B virus (DHBV), which is widely used as a model to study hepadnaviral replication, causes acute hepatitis but is rarely associated with significant histological changes in the livers of chronically infected ducks [35].

## 5. Conclusions

In conclusion, while the significance of the detection of circulating DCH DNA in dogs requires further investigation, the etiology of CH or HCC arising in dogs is unlikely to involve a hepadnavirus.

## Figures and Tables

**Table 2:** Characteristics of a hospital population of dogs in Hong Kong from which whole-blood DNA was tested for domestic cat hepadnavirus via qPCR.

Age in Months (*n* = 501)				Sex (*n* = 499)				Breed (*n* = 501)	
Median	Minimum	Maximum	Interquartile Range	Male		Female		Pure Breed	Mixed Breed
				Neutered	Entire	Neutered	Entire		
122	3.5	226	75	216 (43.3%)	67 (13.4%)	195 (39.1%)	21 (4.2%)	456 (91%)	45 (9%)

## Data Availability

Not applicable.
